# An adaptive spark-based framework for querying large-scale NoSQL and relational databases

**DOI:** 10.1371/journal.pone.0255562

**Published:** 2021-08-19

**Authors:** Eman Khashan, Ali Eldesouky, Sally Elghamrawy

**Affiliations:** 1 Department of Computers and Systems, Faculty of Engineering, Mansoura University Mansoura, Egypt; 2 Department of Computer Engineering, MISR Higher Institute for Engineering & Technology, Mansoura, Egypt; University of Pisa, ITALY

## Abstract

The growing popularity of big data analysis and cloud computing has created new big data management standards. Sometimes, programmers may interact with a number of heterogeneous data stores depending on the information they are responsible for: SQL and NoSQL data stores. Interacting with heterogeneous data models via numerous APIs and query languages imposes challenging tasks on multi-data processing developers. Indeed, complex queries concerning homogenous data structures cannot currently be performed in a declarative manner when found in single data storage applications and therefore require additional development efforts. Many models were presented in order to address complex queries Via multistore applications. Some of these models implemented a complex unified and fast model, while others’ efficiency is not good enough to solve this type of complex database queries. This paper provides an automated, fast and easy unified architecture to solve simple and complex SQL and NoSQL queries over heterogeneous data stores (CQNS). This proposed framework can be used in cloud environments or for any big data application to automatically help developers to manage basic and complicated database queries. CQNS consists of three layers: matching selector layer, processing layer, and query execution layer. The matching selector layer is the heart of this architecture in which five of the user queries are examined if they are matched with another five queries stored in a single engine stored in the architecture library. This is achieved through a proposed algorithm that directs the query to the right SQL or NoSQL database engine. Furthermore, CQNS deal with many NoSQL Databases like MongoDB, Cassandra, Riak, CouchDB, and NOE4J databases. This paper presents a spark framework that can handle both SQL and NoSQL Databases. Four scenarios’ benchmarks datasets are used to evaluate the proposed CQNS for querying different NoSQL Databases in terms of optimization process performance and query execution time. The results show that, the CQNS achieves best latency and throughput in less time among the compared systems.

## Introduction

**Fig 1:**
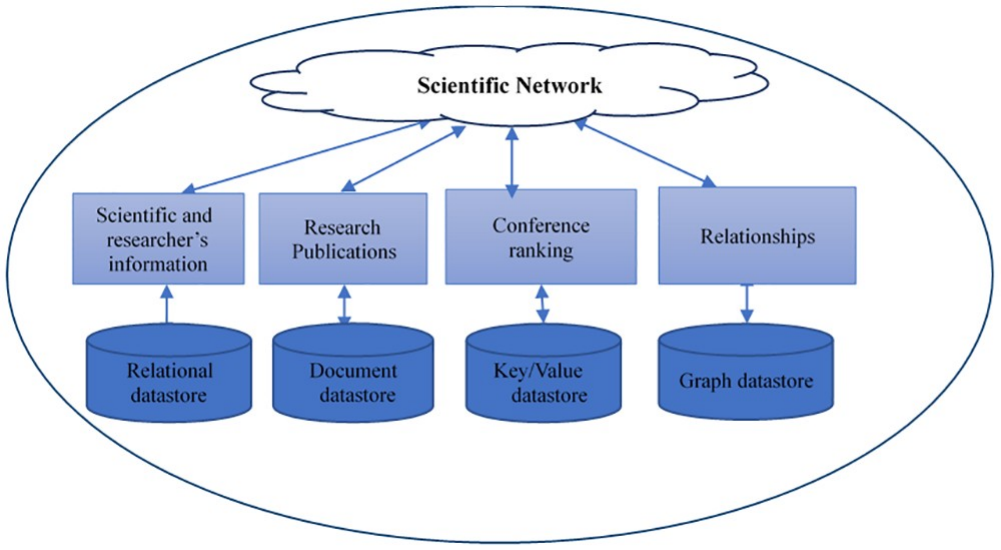
Connecting heterogenous data sets to social network.

The popularity of NoSQL systems is caused by their efficiency in handling unstructured data and backing up effective design schemes that give the system users supreme flexibility and scalability. This paper identifies a relational database and several categories of NoSQL Databases with structural features: key-value, graph, column and document databases. Likewise, every NoSQL database has a special query language and does not support the criteria of other systems. The main problem that many researches focused on, is that there is no standard method to execute complex queries across NoSQL Databases [1–8]. Currently, data stores have several diversified APIs. The programmers of applications based on multiple data stores must be familiar with these APIs during the process of coding these applications. As a result of the variety and changes in the data models [9] of various databases, there is no standard way to solve the problem of implementing queries for various NoSQL data stores. The reason is due to a lack of a combined access model for diversified data stores. The programmers must challenge themselves with the execution of these queries, which are hard to optimize. On the other hand, optimization puts certain criteria into consideration, such as data transformation and movement costs, which might be expensive for big data. Fig 1 shows a diagram of integrating heterogenous relational and NoSQL datasets to an example of scientific social network.

All of these reasons encourage sharing in the interoperability between two or more varied and powerful frameworks. In this paper, Mongo and Cassandra focus on being the most popular way to help companies make business decisions. Several researchers and developers have focused on this problem. The variety of relational and NoSQL data models (relational, key value, ordered key-value, document, semi-structured and graph databases) and query languages (SQL, Cassandra Query Language (CQL), MapReduce querying language, etc.) is the main difficulty. Salami et al. [1–6] Identify a common data model and use algebra to address complex declarative inquiries. In this technique, queries are handled in multiple data stores called VDS (Virtual Data Store), that is, default data stores. The optimization stage is carried out by a two-step broker. First, the selection and project processes are defined down to the local data stores. This allows to reduce the amount of data exchange. Second, an ideal distributed plan is designed with a dynamic programming method. The distributed plan seeks to reduce I / O and CPU costs and to charge and convert data. This technology, however, is restricted to the redress of an ODBAPI query and some query operators. P.Sangat et al. [10] developed another method, named DIMS. In DIMS Most data produced by omnipresent application areas have the character of time series, such as tracking power station data, and from others a pattern of interrelationship emerges, like patient correlation, illness, and symptoms. Additionally, it also features high sampling rate and high data generation rate. A data storage device should have different capabilities to meet the needs of specific requirements, like creating different systems and settings for various applications. Song et al. [11] present Haery, a column dedicated to big data, architecture, implementation and assessment. Haery is developed on Hadoop HDFS and MapReduce based distributed computing platform. The most stable and effective results of Haery’s download and query performance are. But, as data volume increases, there is more cost in time. The following models and algorithms were proposed by Haery: Key-Cube, an improved Z-order linearization algorithm and address tree, Accumulation, a key-cube development method, Query methods to implement key-cube and physical storage queries [12] and system architecture, components and execution of Haery. MusQ, a method of multi-store queries suggested by Ramadan, Hani and others [13], uses a structured approach. Three key features are accomplished by MusQ by solving three crucial challenges: (1) constructing a global schema by leveraging relationships from local source systems, as in the federal approach; (2) conducting complicated queries on various data stores without learning all the different database languages; and (3) effectively executing user queries (joining procedures in particular) to obtain relevant data. In this paper, a Complex Query SQL and NoSQL (CQNS) framework (CQNS) is a proposed for improving and estimating complex queries for relational databases and other types of NoSQL data stores. For this purpose, a unified data model is proposed that uses a suitable environment such as Apache Spark with MongoDB [14] to optimize the qualification of the data ingestion process over Mongo and MySQL databases.

None of these researchers offers an automatic, unique, and fast way to solve these kinds of simple and complex database queries. This paper presents an architecture for automatically solving simple and complex queries, optimization, and evaluation called CQNS. In this proposal, the matching selector layer automatically selects and routes queries to the SQL or NoSQL database engine after performing the matching process on at least five queries coming from user file. Hadoop and Spark environment were also used on the processing layer and query execution layer to speed up the time of executing queries in the correct engine. The main features of the proposed solution are (1) suitable for real implementation, (2) does not require complex mathematical equations, (3) achieved the best and fastest performance, (4) achieved high accuracy compared to other systems. The advantages and disadvantages between the proposed framework and the most recent comparatives solutions are presented in Table 1.

**Table 1:** The advantages and disadvantages of the proposed framework and the most recent frameworks.

Literature	Advantages	Dis-advantages
ODBAPI [1–6]	It uses a mediation-based component to optimally execute complex queries over multiple data stores in Cloud environments.	It took into account the cost-performance model but did not care about the execution time of queries when trying to solve this problem
DIMS [10]	DIMS offers an integrated analysis environment using MongoDB and Apache Spark. It uses the official MongoDB Spark connector to evaluate the performance of MongoDB in sharing and non-sharing.	DIMS did not support complex queries and also did not support most NoSQL databases.
Haery [11]	Haery is a column dedicated to big data, architecture, implementation and assessment. Haery is developed on Hadoop HDFS and MapReduce based distributed computing platform. The most stable and effective results of Haery’s download and query performance are.	It is noticeable from this system that with the increase in the amount of data, the execution time increases significantly.
MusQ [13]	MUSQ is framework for performing complex queries on multiple data stores using Datalog query language.	It Support only MQL queries. It uses more complex equations when try to solve this problem.
Proposed framework (CQNS)	CQNS is suitable for real implementation. also, it does not require complex mathematical equations. CQNS use hadoop and spark to speed up the timing of execution.it achieved the best and fastest performance and achieved high accuracy compared to other systems.	There are some NoSQL database libraries that have not been added to the system and will be added in future work

The rest of this paper is organized as follows. In section 2, this paper presents the related work. In section 3, the proposed CQNS framework, which has three layers, is presented. In section 4, the implementation and evaluation of CQNS is discussed. Section 5 provides conclusion and future work.

## Related work

Several researchers and developers have focused on solving simple and complex queries over heterogenous datastores problem. The variety of relational and NoSQL data models (relational, key value, ordered key-value, document, semi-structured and graph databases) and query languages (SQL, Cassandra Query Language (CQL), MapReduce querying language, etc.) is the main difficulty. G. Baruffa et al. [15] and Fjällid J. A [16] are characterized a Spectrum Sensing that provides a service which allow end users to easily access and process wireless spectrum data. To reduce the latency of services provided by the platform, that adjust the data processing chain, they took an interest in Mongo and Cassandra databases and did not consider the rest of the databases. Khan et al. [7]. and Duggan et al. [17] it offers frameworks that called PolyWeb and BigDAWG, respectively. PolyWeb and BigDAWG retain data sources in a primary format, that is, without serializing them in a common data format. In PolyWeb, SPARQL queries [18] are translated into the original query language for these sources. PolyWeb indexes each data source to predict the query and creates deep left plans. Despite the efficiency, the current methods are not able to exploit knowledge about the main features of integrated data sources, and produce custom query plans for selected sources to collect data from the data lake. In contrast, the QODM [19] approach produces distinct schema using the data model and data schema of an application for NoSQL Databases. This approach will not prevent programmers from using any NoSQL database. Document and relational data stores are integrated in a hybrid mediation approach proposed by the authors in [20, 21]. However, these frameworks don’t take into account the rest of NoSQL data-stores. The articles [13, 22]. presented a mediation platform based on rewriting queries. A semi-structured data model called OEM was suggested by Tsimmis [22] and grants support for several data stores using the global schema and related query language (Lorel). In Lorel, a global schema is used to rewrite queries, and this method is considered a view approach for the data sources, but lacks query optimization. The articles [23–25] are studying the Performance and Scalability of querying large-scale heterogeneous models but they got unexpected results in case of neo4j as it took longest time as compared to MongoDB and PostGre SQL. IBM NoSQL [26] is a commercial solution that permits a database for storing SQL and NoSQL data in the same data-store. A problem is that this solution does not support accessing the database from outside of IBM servers. S. K Pandey et al. [27] presented the CBCQL framework, which is internally mapped to CQL and so has the same power as Cassandra, but this framework does not support other NoSQL Databases. A relational database is moved to Apache Cassandra by designing a data model of the application designed by the MySQL database by Aaron Schram and Kenneth M. Anderson [28]. This design does not explain the process of implementation for other applications. The article from Ferro et al. [29], has been discussed using the document-oriented NoSQL database in GDW design. In this study, the focus was on determining the frequency level of geospatial data that provides low storage cost and good SOLAP query performance. In this sense, UML diagramming modeling has been codified in the MongoDB database. However, other types of databases were not tested. Agrawal et al. [30] and Sánchez-de-Maradiaga [31] designed a model to measure the performance with regard to spatial databases and to see whether NoSQL performance was better than SQL. It studied Mongo database as an example of the NoSQL database, but did not study the other databases. The authors in [32–34] trying to extend the features of the EtherQL query by allowing multiple search parameters and a number of analytical functions. They concluded that their implementation was less productive than Ethereum. However, few NoSQL Databases are supported only by these frameworks, so the programmer has to make designs for data models of an application and choose a proper strategy for data mapping. The authors in [35] presented a model to study the performance of querying in NoSQL and relational databases with MongoDB and MySQL. Santana LH et al. [36] presented an analysis of mapping strategies for storing RDF data into NoSQL databases. I. Mearaj et al. [37] presented a framework based on the Mongo database engine, which works to convert the form of data from tables to unstructured documents depending on the features within the Mongo engine such as its compatibility with other programs. R. Gunawan et al. [38] presented a framework that comparing the execution time of CRUD queries. MongoDB, Arango DB, and CouchDB were experimented with by processing queries for repeated create, read, update, and delete commands in different quantities. Khashan EA [39] provides a framework that can handle complex queries over sql and NoSQL databases using Mongo and cassandra data stores. CQNS is a proposed framework for improving and estimating complex queries for relational databases and other types of NoSQL data stores. For this purpose, a unified data model is proposed that uses a suitable environment such as Apache Spark with MongoDB [40–42] to optimize the qualification of the data ingestion process. The CQNS framework transforms each query process received from any dataset to the matched Engine after using Hadoop/HDFS and Hadoop/MapReduce with parallel k-means clustering for processing data without physical transformation data.

## Proposed CQNS framework

**Fig 2:**
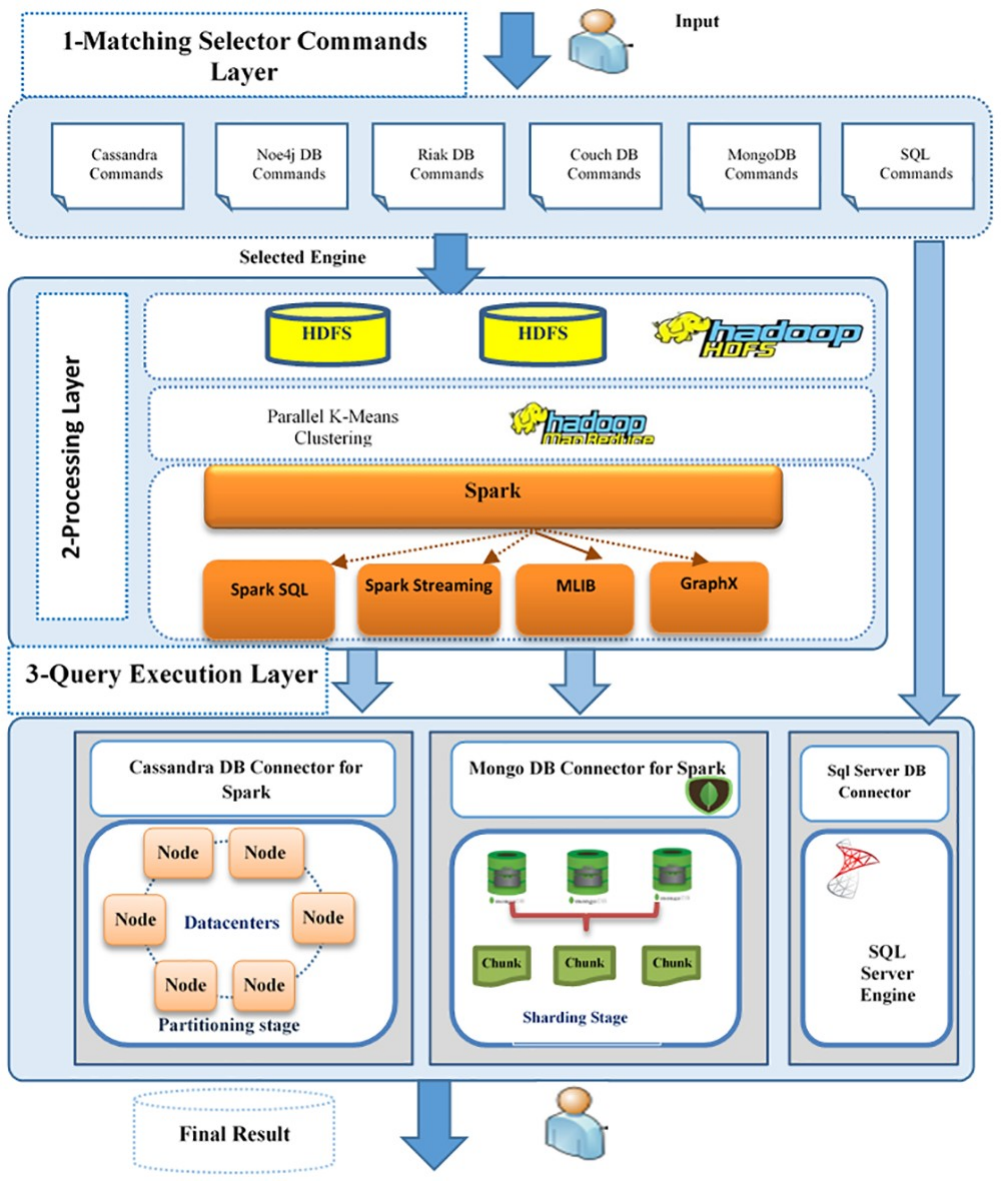
The proposed framework (CQNS).

This section introduces the proposed CQNS approach, which is capable of executing complex queries across heterogeneous data stores. This framework consists of three stages, as shown in Fig 2: Matching Selector stage, processing stage, and query execution stage. In the following sections, this paper discusses the different stages of the proposed CQNS framework.

### Matching selector stage

**Fig 3:**
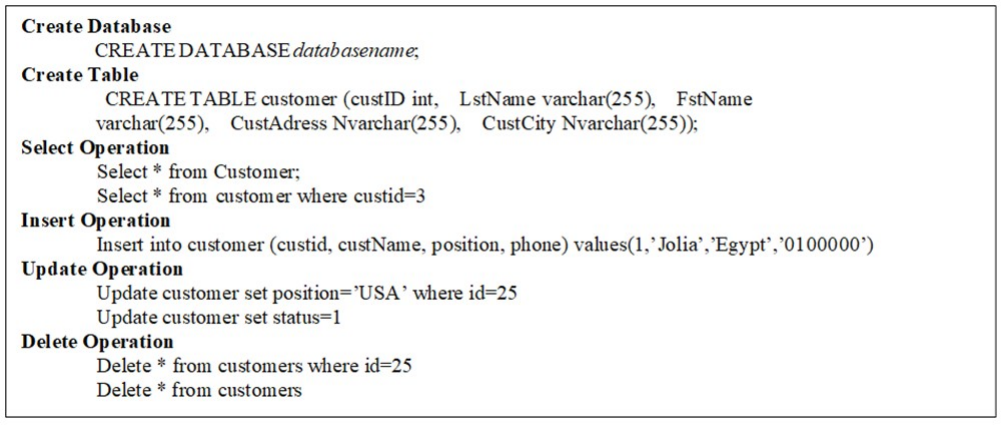
SQL libraries.

**Fig 4:**
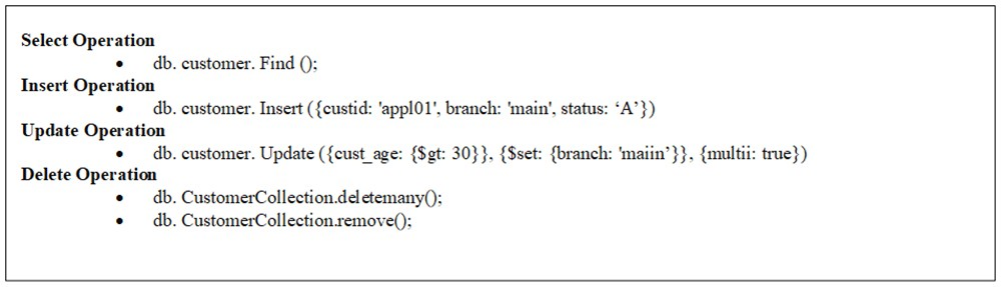
Mongo libraries.

**Fig 5:**
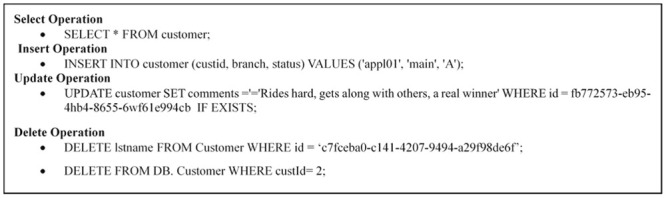
Cassandra libraries.

**Fig 6:**
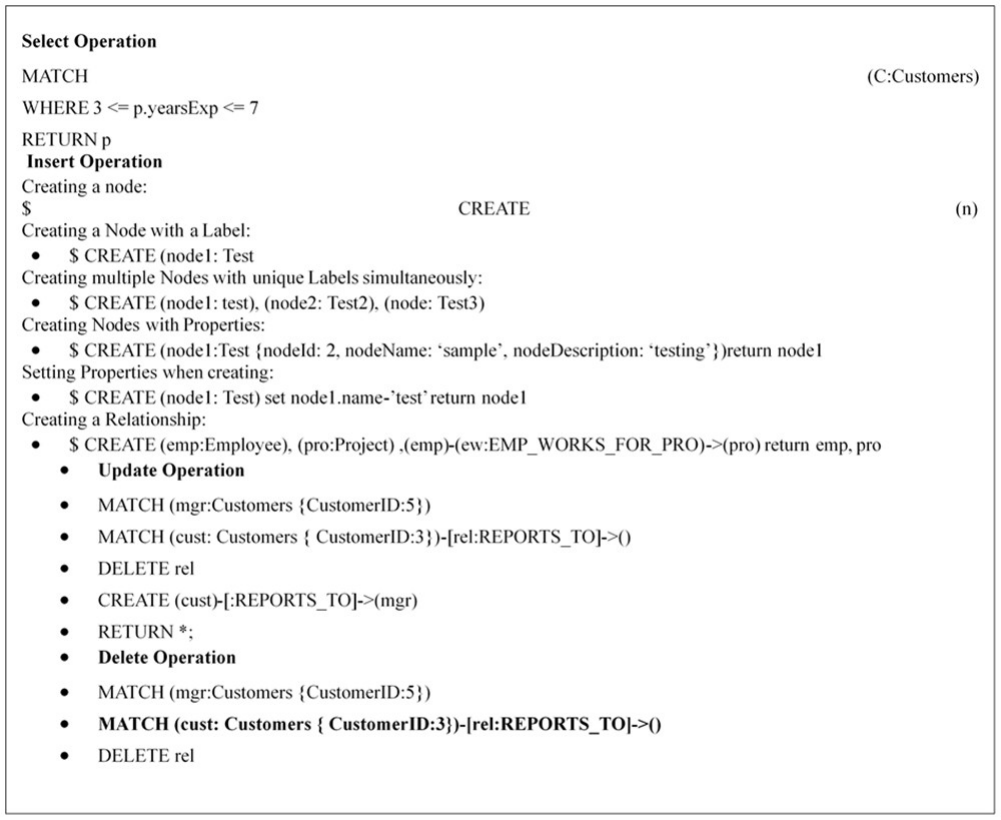
Noe4j libraries.

**Fig 7:**
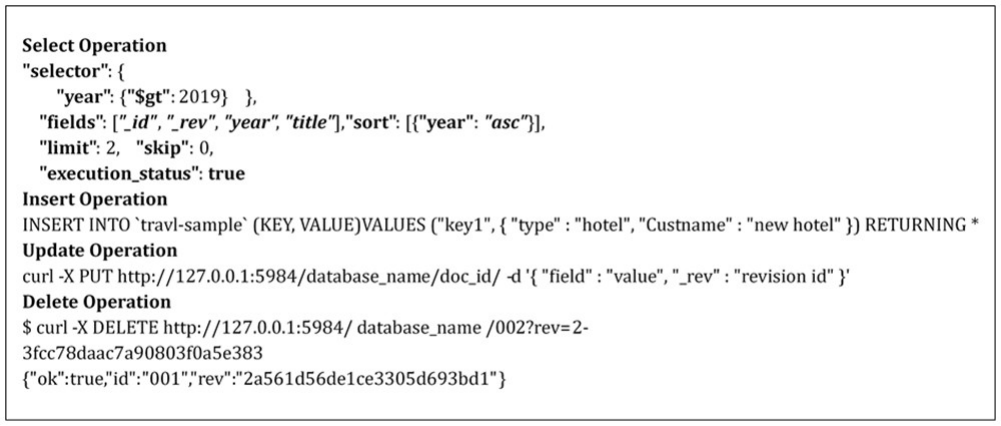
Couch libraries.

**Fig 8:**
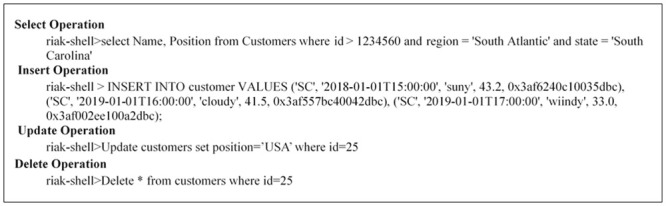
Riak libraries.

This stage receives any SQL or NoSQL database query to match the sentences of the query given by the user with the stored libraries that hold a number of statements for each database type either SQL or NoSQL from the database engine and then compares the sentence with the stored libraries to define the required database engine. This paper prepared a set of libraries for each of the databases that are studied, such as SQL as an example of relational database and MongoDB, Cassandra, Couch, Riak and NOE4J as an example of NoSQL Databases. Indeed, this approach symbolizes the combined parts among every deployed data storage and delivers a unified model to the following stage of the framework. This model contains the particular operations of every database. It is noteworthy that the user has to add a particular implementation of the data store if he/she needs to integrate an extra database. In the following figures, an explanation is given for testing the query statements for the databases used. This paper used SQL Server (as an example of a relational database), MongoDB and Cassandra (as examples of NoSQL Databases). Fig 3a explains the stored SQL libraries statements for SQL database while Figs 3–8 explain the CRUD statements for MongoDB and Cassandra DB respectively, as examples of the NoSQL Database libraries used in this paper.

Algorithm 1 illustrates the method of discovering the database type of the query to be executed based on the libraries stored in the application to show the selected engine database.

According to Algorithm 1, the results of matching patterns and input values, one of the following decisions will be followed:

**Algorithm 1: Matching Selector Algorithm**

Input: *qs* query Statement

Output: *SQL or NoSQL* database Engine

 1. parsing Query Statement (*qs*).

 2. Declare *arr[6]* = {sql, Mongo, Cassandra, riak, Neo4j, Couch}

 3. For *i* = 0 to 5

  {

  if(*arr[i]* = *qs*) selected_Engine = arr[i]

  *break*

  }

 4. Switch selected_Engine

  Case sql

   Connect to sql Engine

  Case Mongo

   Connect to Mongo Engine

  Case Cassandra

   Connect to Cassandra Engine

  Case riak

   Connect to Riak Engine

  Case Neo4j

   Connect to Neo4j Engine

  Case Couch

   Connect to Couch Engine

  Case else

   No Engine

  End switch

 5. If selected_Engine = ‘Noengine’, then display error page and stop running,

  else continue running & execute query qs.

If the patterns are the same as the SQL database the application will continue running the SQL database route. The code can continue to operate the Mongo database route if patterns are similar to the Mongo database. If the patterns are appropriate for the Cassandra database, the path for the Cassandra database will be followed.

If you want to apply patterns to other databases, you must add their own libraries.

### CQNS processing stage

Within this section you will shortly find details about technology and the setup process for this study. CQNS deployed and used Hadoop/HDFS [1] to store the incoming data. Hadoop is an open-source distributed computing platform that mainly consists of the distributed computing framework MapReduce and the distributed document system HDFS [15]. The formula (1) uses to calculate HDFS node storage (H) required:

H: denoted the HDFS node storage required
C: is the compression ratio and completely depends on the type of compression used and size of the data.
R: It is the replication factor which is 3 by default in production cluster.
S: S denotes the initial amount of data you need to move to Hadoop.
I: I represent the intermediate data factor which is usually 1/3 or ¼. It is Hadoop’s intermediate working space used to store the intermediate results of different tools like Hive [43], Pig etc.
1.2: 1.2 or 120% more than the total size.

$$H=\frac{C*R*S}{\left(1-I\right)*1.2}$$ (1)

MapReduce [44, 45] is a software platform for parallel processing programming of large-scale data pieces. The MapReduce strategy is applied to the k-means clustering algorithm and clustered for the data factors. The k-means [46] algorithm can be successfully parallelized and clustered on hardware resources. MapReduce can be utilized for k-means clustering. The results also show that the clusters shaped using MapReduce are similar to the clusters produced using a sequential algorithm. Once HDFS takes data, this process breaks information down into separate blocks and distributes those blocks to different nodes in the cluster, thus enabling high-efficiency parallel processing. The data from HDFS is accessed by a Spark streaming program for handling before being stored in MongoDB in the server of the database. Resilient distributed datasets (RDDs) are an abstraction presented by Spark [28]. RDDs symbolize a read-only multiset of data objects divided into a group of machines that continue operating as designed despite internal or external changes (fault-tolerant way). Spark is considered the first system of programming languages in general and is used as an interactive way to handle big data sets for clustering. A Complex Querying over NoSQL Databases Algorithm (CQNSA) using MongoDB and the MongoDB Connector for Spark is proposed using an open-source NoSQL database that is designed for high scalability, effectiveness, and availability. This CQNSA is shown in Algorithm 2.

**Algorithm 2**. CQNSA Algorithm

1: **Input** qu: A query

2: **Output** schedule: The optimal execution schedule of the query qu

3: selectAttributes ← Ø           # Parsing the SELECT clause

4:  **while** (exist (attribute Att in the clause SELECT)) **do**

5:   select_Attributes:insert(entity_Set_Of(Att); Att)

6: **end while**

7: initNodes ← Ø            # Parsing the FROM clause

8: **while** (exist (entitySet ES in the clause FROM)) **do**

9:  initNodes:create_Pushdown(ES; select_Attributes:get(ES))

10:  **end while**

11:  Join__Conditions← Ø # Parsing the WHERE clause to identify distributed joins

12:  **while** (exist (condition Con in the clause WHERE)) **do**

13:   entity_Set_Left ← site_Of_Left_Entity_Set (Con)

14:  _site ← _siteOf(_entity_Set_Left)

15:  **if** _is_Join_Condtion(C) **then**

16:   **if** _site = = _site_Of_ (Entity_Set_Right (Con)) and _conjoin(site) then

17:    _init_Nodes:_merge(_entity_Set_Left; _EntitySetRight(Con))

18:   **else**

19:    _Join__Conditions. Insert (Con)

20:   **end if**

21:  **else**

22:  _init_Nodes. _insert__Restriction__Condition (entity_Set_Left; Con)

23:  **end if**

24: **end while**

25:  schedule ←_init_Nodes

26:  **for** *i* = 1 to _number of _Join__Conditions **do**

27:  **for each** _condition Con in _Join__Conditions **do**

28:   **for each** schedule s1 in schedules **do**

29:    **for each** schedule s2 in schedules **do**

30:     **if** _match (s1; s2; Con) **then**

31:      _new_Conditions. _insert(_conditions_Of(s1); _conditions_Of(s2); Con)

       # Inserting the condition to a new join

32:      _node_createVDS_Join (s1; s2; Con)

33:      Schedules. Insert(node)# Creating a new schedule with a VDS join node

34:      **if** canJoin(siteOf(s1)) **then**

35:       _node ← create_External__Join (s1; s2; Con)

36:        Schedules. insert(node)

         # _Creating a new schedule with an External join node in s1

37:      **end if**

38:      **if** can_Join(site_Of(s2)) **then**

39:       node ← createExternal__Join (s2; s1; Con)

40:       schedules. insert(node)

        # Creating a new schedule with an External__ join node in s1

41:      **end if**

42:     **end if**

43:    **end for**

44:   **end for**

45:  **end for**

46:  prune(schedule)      # Pruning non optimal and redundant sub- schedule

47: **end for**

### Query execution stage

Instead of storing the data as tables with columns and rows, the data are stored as documents. Every document can be one of the relational matrices of the numerical values or the overlapping interrelated arrays or matrices. These documents are serialized as JSON objects and stored internally using JSON binary encryption known as BSON in MongoDB; the data is partitioned and stored on several servers called shard servers for simultaneous access and effective read/write operations. MongoDB and Apache Spark are integrated seamlessly by this connector. MongoDB aggregation pipelines and a problem of how to assign a group of objects into groups, called blocks, so that the objects within the same group, partitioning is by using a cluster assignment function *C*: *X* → {1, 2, …., *k*} when X is a set of objects, the Number of clusters $K\in Z^{+}$ and Distance function d∈R0^+ between all pairs of objects in X, partition X into K disjoint sets *x*1, *x*2, .…., *x*_*k*_ such that $\sum_{k}\sum_{x,x^{'}\in X_{k}}d(x,x^{'})$ With N = |X|, the number of distinct cluster assignments possible as follows [47]:

SN,K=1K!∑k=1K-1K-kKkkN (2)

#### MongoDB engine

**Fig 9:**
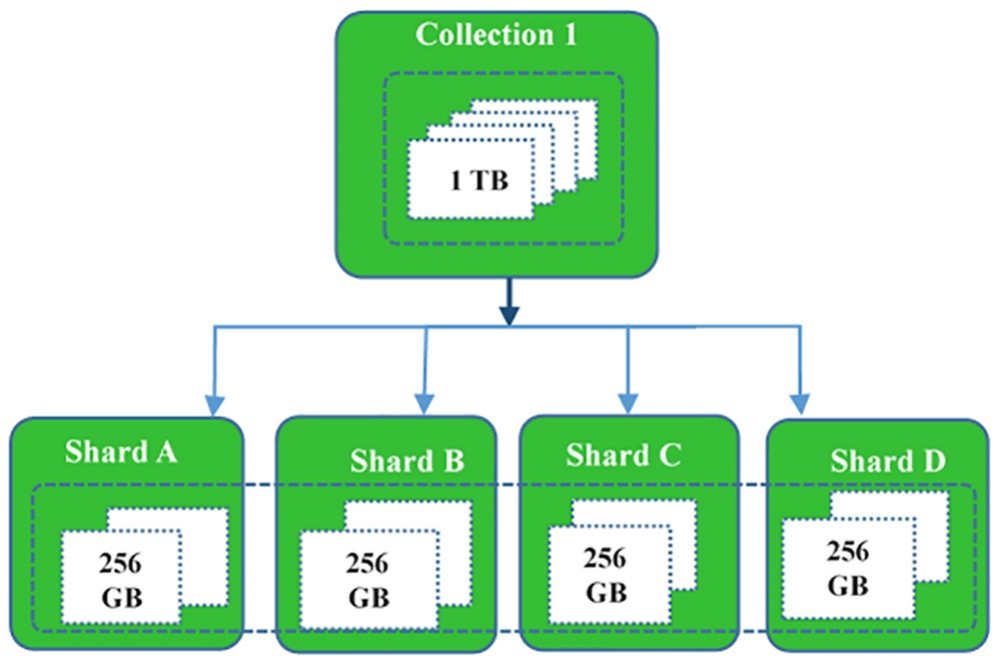
Sharding Mongo DB Stage.

Sharding is a way to distribute data across multiple devices. This work provides MongoDB which utilizes Sharding to benefit implementations using very huge databases and structures that are highly efficient. Data stores which contain large datasets or high-productivity applications may challenge a single server ’s ability. High query rates for example can exceed the server’s CPU capability. A number of sizes greater than the RAM of the device will help validate driver I / O capability. A database may have a mix of sharded collections and unshared collections. Sharded sets are divided into a cluster and spread throughout the shards. Unshared collections on a main shard are stored. As shown in Fig 9 each database has its own main shard.

The Mongo DB uses the following equations to measure the theoretical maximum collection size. This Study supposes *M* is the max Splits, *md* the maximum BSON document size is 16MB or 16777216 bytes. mb is the maximum collection size(mb), *C* is the chunk size, and *avg* is the average size of shard key values in bytes [48].

$$M=\frac{md}{<avg>}$$ (3)

[48]

$$MB=M*\frac{C}{2}$$ (4)

[48]

**Fig 10:**
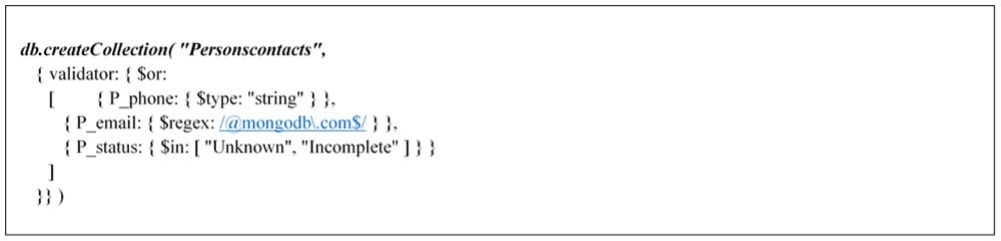
Json example for specifying validator rules.

In addition to JSON schema validation, MongoDB manages validation with query filter expressions using query operations, with the exceptions of $near, $nearSphere, $text, and $where. Fig 10 explains a JSON example of specifying validator rules using the following query expression:

#### Cassandra engine

**Fig 11:**
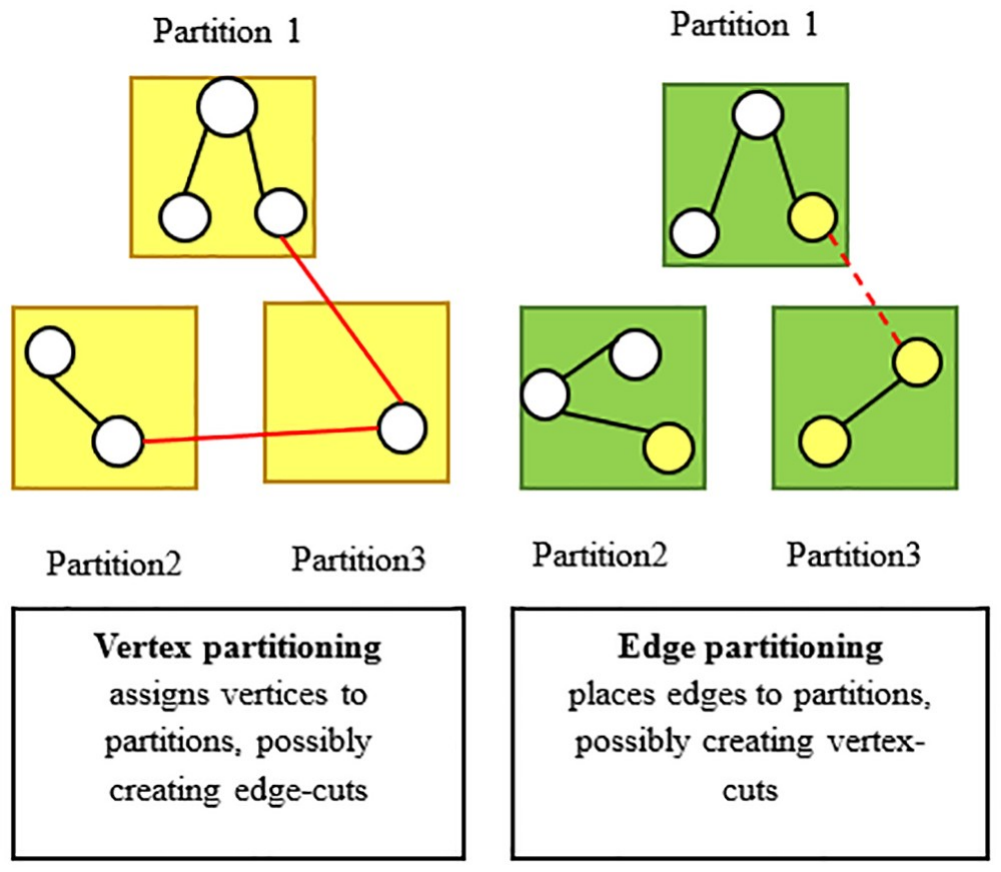
Vertex partitioning and edge partitioning in the Cassandra database.

The Apache Cassandra database has linear scalability and proven tolerance for hardware or cloud infrastructure, and these attributes make this database an ideal platform for important data. This paper presents replication supported by the Cassandra database across multiple data centres that is best in class, providing less downtime for users and peace of mind by knowing that it can overcome regional interruptions. This paper proposes two kinds of partitioning methods that can work with the Cassandra database: vertex partitioning and edge partitioning. Later, this study will introduce how can research paper dealing with these methods. This paper investigates vertex partitioning and edge partitioning to show differences in the results about them, as shown in Fig 11.

#### Data partitioning

Cassandra divides the database into smaller, partially overlapping datasets that are stored locally on each node. Thus, unlike other NoSQL Databases such as HBase, Cassandra does not require a shared file system (for example, HDFS). A hash function is used to distribute basic registry keys for the nodes. This process is performed by dividing the scope of the hash key into subdomains called partitions (also called token ranges). In blocks without repeating (RF = 1), each node can be configured to store unique partitions locally. In this section, the necessary background will be provided and presented with the data and account models we target. Table 2 contains the partitions variables used in this paper. This paper uses formula (5) to calculate the size of data partitions [49].

$$NV=Nr\left(Nc-NpK-Ns\right)+Ns$$ (5)

**Table 2:** The partitions variables used in this paper.

Symbol	Description
*Nv*	The number of values (or cells) in the partition
*Nr*	The number of values per row.
*Nc*	the number of columns.
*NpK*	the number of primary key columns.
*Ns*	The static columns.
*St*	The size of partitions.
*Ck*	partition key columns.
*Cs*	static columns.
*Cr*	regular columns.
*Cc*	clustering columns.
*tavg*	the average number of bytes of metadata stored per cell, such as timestamps.
*sizeOf()*	This function refers to the size in bytes of the CQL data type of each referenced column.

In order to determine the size, this study uses formula (6) Scale St of a partition for assessing [49]:

$$St=\sum_{i}SizeOf\left({Ck}_{i}\right)+\sum_{j}SizeOf\left({Cs}_{j}\right)+Nr\times \left(\sum_{k}SizeOf\left({Cr}_{k}\right)+\sum_{L}SizeOf\left({Cc}_{l}\right)\right)+SizeOf\left(t_{avg}\right)\times Nv$$ (6)

## CQNS evaluation

CQNS is used to store, manage and execute bigdata queries and makes the development job very much easier. In this paper, the proposed model rewrites each query into the particular query language of the integration data store. The processing stage in CQNS turns results into a suitable format such as JSON before responding to the system users. Therefore, the overhead is considered reasonable to some extent. Because of memory management trouble in the driver, there is a probability that the performance of CQNS will degrade after 50000 entities. The results of experiments testing MongoDB and Cassandra DB are shown in the following sections.

### Datasets

To assess the suggested framework strategy for querying NoSQL Databases, the research work performed experimental tests using four scenario benchmark datasets for hybrid datasets. A description of the dataset used in this paper can be downloaded from http://snap.stanford.edu/data/. All of the datasets are related to complex querying. The datasets used in our research have a large number of features (thousands of rows) that are suitable for displaying the efficiency of our strategy for high-dimensional data. The ego-Facebook, ego-Twitter and soc-LiveJournal1 datasets were acquired from different types of database engines.

### System analysis details

This section gives a brief overview of the technologies and preparation for the study. This research develops then began publishing the preceding environment requirements: Hadoop, HDFS, Apache Spark, MongoDB, Spark, Cassandra DB, Spark server Cassandra DB, SQL.

1. Hadoop / HDFS Setup: This paper deploys Hadoop / HDFS version 2.10.0 using standard configuration parameters. The application server is running on HDFS to store incoming data and access software Spark Access data from HDFS for processing before inclusion in SQL, MongoDB and Cassandra DB in the database server.
2. Spark Setting: Apache Spark is a largely open-source environment. Spark introduce a stripping known as elastic Distributed Data Sets (RDDs), representing a number of read-only data items split into a range of tolerant devices. Spark is the first system that uses a general programming language interactively in order to collaborate with large data sets on a block. For standalone mode and control configuration of the application code, a version of the Apache Spark 2.3.2 is available. For instance, spark.serializer was set as org.apache.spark.serializer. KryoSerializer by the experiments. Using Scala 2.10.4 was built by this experiment.
3. Database Connector for Spark: This connector provides a seamless integration between matched Database and Apache Spark. It makes effective use of database assembly lines and secondary indexes to extract, filter and process the sub-data required for the Spark process. Additionally, for maximizing performances over a huge distribute Datasets, they link the RDDs to the source database node and reducing the data transfer across the cluster.
4. Hardware: The Spark app server has 16 dedicated hubs, 64GB of memory, 459GB of hard drives, and 64-bit Ubuntu GNU / Linux. The mongo dB database server and both Shard 4 server have dedicated cores, 16GB memory and 130GB HDD, while each of the initialization servers contains 1 hard disk, 4GB and 30GB. The Cassandra database server. 16 GB memory, 130 GB hard drives, and Microsoft SQL 2017 server has been installed with the same infrastructure specifications previously mentioned.

### Cost model

The cost of implementation is the sum of the costs of each process that composes the implementation plan. This is worth noting that costs do not reflect time directly. More cost means more time, of course. It is used to compare two question execution plans, but not for estimating the responding time directly. The multiplication of the matrix between the *α*, *β* and *α* coefficients row was determined to test the expense formulation according to each data store. A column vector includes the values of the catalog parameters and a fixed variable known as const, which is a scale and may be a cardinality, a number, etc. Furthermore, the value of the column vectors will be empty if the parameter is not based on a particular calculation (CPU cost, I / O cost or the costs of connections). This is determined as follows: Matrix multiplication [1]:

$$const\left(\alpha \;\beta \;\gamma \right)\left(\begin{array}{c} t_{cpu}\\ t_{i/o}\\ t_{conn} \end{array}\right)=const\left(\alpha \times t_{cpu+}\beta \times t_{i/o+}\gamma \times t_{conn}\right)$$ (7)

Where *t*_*cpu*_ symbolized the CPU time, *t*_*i/o*_ symbolized the Input /Output time and *t*_*conn*_ symbolized the connection time to engine.

The *α*, *β* and *α* are represents the coefficients that were considered to evaluate the cost model. These of parameters are separately defined to each data store, and a constant variable called *const* which is a scalar and can be a cardinality, selectivity, etc.

The projection cost and selection processes that are referenced by *costProjcton* and *CostRestrction* are started with forms respectively (see formula 7 and formula 8). The operating cost is the linear mix of variables for *initProjcton* (*resp*. *InitSelection*) and scanning and dropping (resp. selection). As the input variable *N*, the *costProjcton* indicates the integrated data store in which the connection is executed, variable *H* indicates the sum of the entity size specified in the entry, and the estimate function named *estm*. This function allows to measure how much the primary projection (*resp*. *selection*) is carried out. The range and cardinality variables are referred to:

n: *a node*, h: *length of an entity set*, *projatt*: *Projection attributes*

$$cstproj\left(n,h,estm\left(projatt,h\right)\right)=initproj\left(n\right)+scan\left(n\right)*h+proj\left(n\right)*estm\left(projatt,h\right)$$ (8)

[1]

*n*: a node, *h*: length of an entity set, *restcond*: restriction condition

$$cstRest\left(n,h,estm\left(restcond,h\right)\right)=initrest\left(n\right)+scan\left(n\right)*h+rest\left(n\right)*estm\left(restcond,h\right)$$ (9)

[1]

The cost formula for the linking process, referred to as *costJoin*, is then determined (see formula 10). This has an external connection or a VDS link. This method is a linear mixture of *initJoin* variables for scanning and joining. It takes *costJoin* to specify either an integrated data store or VDS, the variable *h* to indicate the full size of the entry group, and the estimation function we call the estimate. *costJoin* is the input parameter N. The condition and selective variable are included. At best, the estimate’s value must be the same as that of the resulting group of entities. The value of the estimate is equal, in the worst-case scenario, to those of the two joining entities that are originally Cartesian.

*n*: a node, *h*: length of an entity set, *jcond*: join condition

$$costJoin\left(n,h,estm\left(jcond,h\right)\right)=scan\left(n\right)+scan\left(n\right)*h+join\left(n\right)*estm\left(jcond,h\right)$$ (10)

[1]

Performance estimation is an important point for a new framework. This estimation is shown in the outcomes of total cost, average time, and ingestion rate. These outcomes are utilized to estimate the efficiency of the proposed framework. Table 3 introduce the main parameters that used to define the cost model of the proposed framework. The outcomes are calculated by the following equations:

$$TotalCost=\alpha \times t_{cpu+}\beta \times t_{I/o+}\gamma \times t_{cconn}$$ (11)

[1]

$$Averagetime=\frac{Totalcost}{No.ofjoins}$$ (12)

[10]

$$Ingestionrate=\frac{No.ofrecords}{Averagetime}$$ (13)

[10]

**Table 3:** Specification of the cost model parameters.

Symbol	Specification
α, β, γ	are represents the coefficients that were considered to evaluate the cost model
t_cp_	defines the CPU cost
t_i/o_	defines the Input /Output cost
t_conn_	symbolized the connection time.
cstproj	Cost formula of the projection operation
cstRest	Cost formula of the selection operation
conjoin	parameter that denotes if a data store supports joins or not
costJoin	This parameter used to specify either an integrated data store or VDS.
n	This parameter defines the node in the execution plan.
h	This parameter defines the length of an entity set.
jcond	This parameter defines the join condition
Estm (projatt, h)	Defines the value of the estimation.
Initrest	Represents the total time of allocating the node to execute queries
Initprog, scan, and prog	These parameters have been used for linear combination in formula 8.
Initrest, scan, and rest	These parameters have been used for linear combination in formula 9.
totalcost	This parameter used to calculate the framework total cost.
Average time	This parameter used to calculate the framework average time according to number of joins.
No. of joins	This parameter defines the number of joins.
Ingestion rate	This parameter defines the Ingestion rate.
No. of records	This parameter Denotes to number of records

### CQNS join queries

The purpose of these experiments is to estimate the impact and validate the proposed optimization process. Thus, various designs utilized to optimize the process are tested to ensure their efficiency and integration. The purpose of this paper is to contrast various strategies to measure both the response time and the latency time precisely and accurately. This purpose is possible due to experiments that are performed in this proposal using two variables of join queries. For variable No. 1, every entity set might join not less than one entity set and not more than two entity sets, creating a linear form, which is generally utilized in several applications. For the second variable, all entity sets executed in a query join a similar entity set, creating a star form, which is widely used in data warehouse applications. The two variables of queries are implemented through four different possibilities. It is necessary to know whether the sub-queries in these possibilities are implemented in a parallel or sequential way and whether external join implementations are enabled or disabled.

- Possibility 1: This will happen when a join query is executed sequentially by using the VDS and integrated data store (most simple possibility) for push down operational activities.
- Possibility 2: The connectors will sequentially use both the VDS and the integrated data store for external connections or the push-down process when executing a joint query.
- Possibility 3: This option is presented when a join query is executed in parallel by using the VDS only. Parallelism is executed in the integrated data store for implementing push-down operations.
- Possibility 4: This option occurs in conjunction with the VDS and integrated data storage when the joint search is performed. The execution of external connections and the Push-down process are paralleled. Furthermore, the experiments are performed with two datasets that are different in size. The first of these datasets is medium-sized, while the second is large-sized. Note that these various sizes are multiplied by hundreds of factors. There are ten entity sets that are different in size, starting from some megabytes and ascending to hundreds of megabytes, used in the experiments and saved on five various types of data stores. The experiments are conducted by proposing to utilize four querying processes, such as linear and star joins, and increasing the join counts for both from three to ten. Moreover, the subsequent constraints on the variables in the catalogue are considered. In fact, the variables in the catalogue are considered. The variables a, b and c shown in Table 4 symbolize CPU timing, input/output timing and connection timing, which are correspondingly assigned to five data stores and the VDS.

**Table 4:** Sample catalogue variables.

Data_stores	*α*, *β*, *γ*	convert	ships	scans	load	Init_ETSL	Cardinality	Init_Join	Join_No
D1	2	6	6	2	2	20	Entity_Set A = 4000000	5	0; 2
D2	2	8	8	2	2	20	Entity_Set B = 1000	20	4
D3	2	2	2	2	2	2	Entity_Set C = 3000	2	2
D4	2	6	6	2	2	2	Entity_Set D = 10000	6	16
VDS	2	2	2	2	2	8	__	10	2

### CQNS linear join experiments

**Fig 12:**
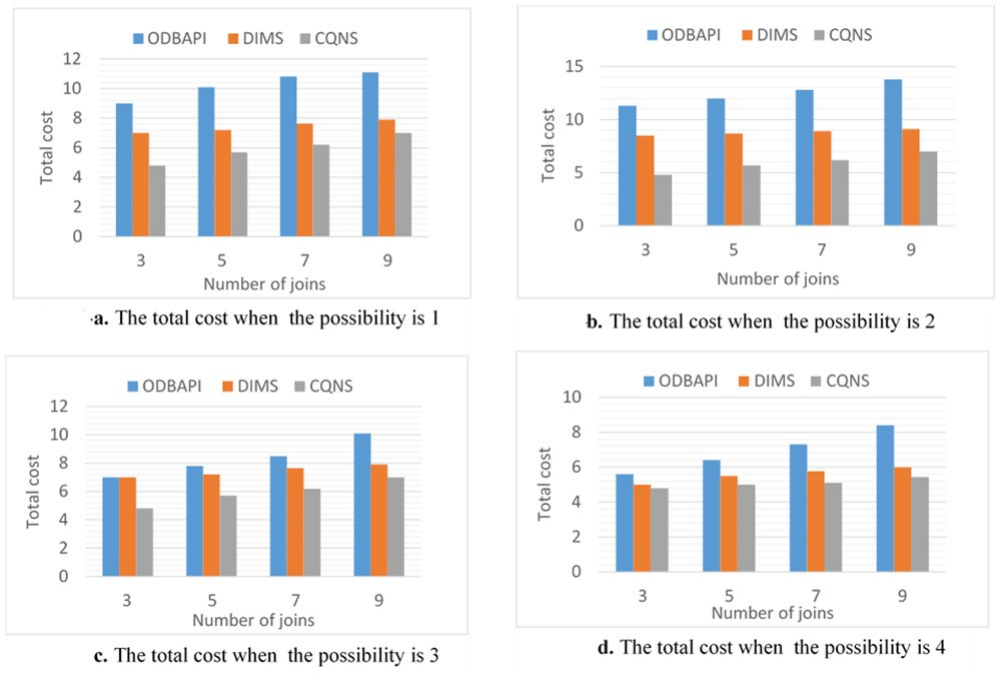
Comparison of possibilities 1 to 4 for ODBAPI, DIMS and CQNS on linear joins.

**Fig 13:**
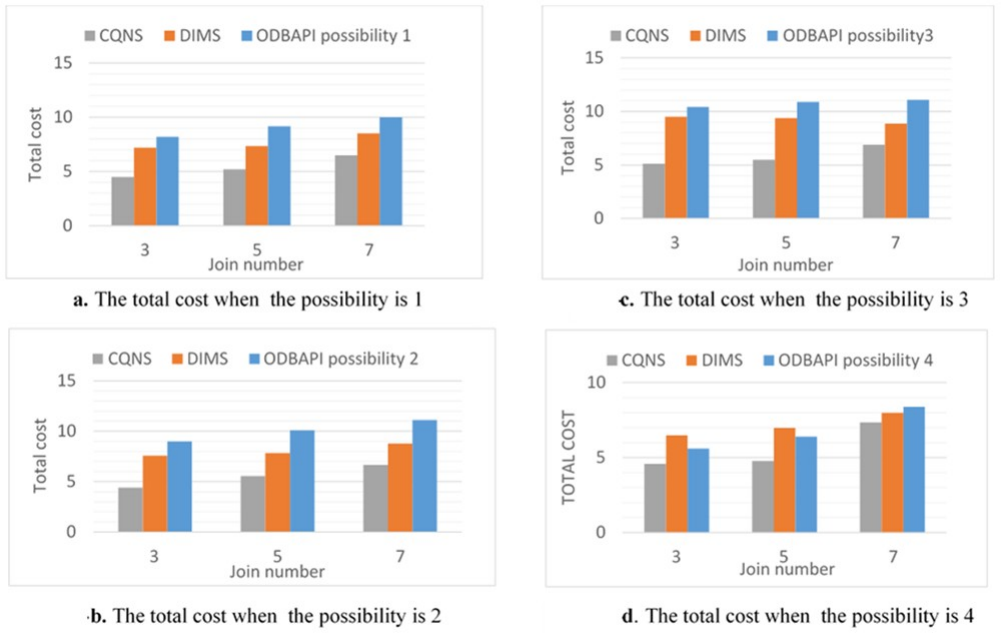
Comparison of possibilities 1 to 4 for ODBAPI, DIMS and CQNS on star joins.

According to possibilities 1 to 4 and the number of joins, the overall time estimations from the time model are presented in Fig 12a and 12b. In Fig 13a and 13b, the results are explained for accessing very large-sized data, such as Big Data-Context. Every cardinality is multiplied by 100. Based on these results, the main aspects of this proposition are proved for creating an optimal implementation plan without dependence on the variable of the join query. The following conclusions have been obtained on the basis of the results shown in Fig 12c and 12d, and Fig 13c and 13d. In fact, the assumption in this paper is that only data stores D1, D2 and D3 support the join query implementation. The integrated data store is then supplied so that one or more set of entities can be saved, e.g., in storage area D2. Note that the number of entities in the suggested entity set for a standard size data set is shown in the chart. The external joins show the following significance: both possibility 2 and possibility 4 indicate the importance of utilizing the integrated data store in the implemented join queries. In fact, a better aggregate cost is obtained in comparison to that of the possibilities that are executed using the VDS. The gain in possibilities 1 and 2 and that in possibilities 3 and 4 are computed to obtain the average gain, which is equal to 92.23%. The importance of a parallelization is this: The simultaneous implementation of external connections and push-down operations has been advantageous and has resulted in a significant gain from the other two (which are sequential to implement). The average increase is 86.67 percent compared with other possibilities.

### CQNS star joins experiments

When possibilities 3 and 4 are applied using star joins, the gain becomes great. The consequence of this is, in actuality, that all compression operations are carried out simultaneously. It is important to integrate various optimization methods as shown: For possibility 4, the work of the integrated data store is maximized, and queries are executed in parallel. The probability of the best average cost in comparison with the simpler cost is 1. in actuality, the mean profit was found to be 99.98%. (Fig 13a–13d) explain the difference results between ODBAPI, DIMS and our framework CQNS.

### CQNS optimization time in MongoDB

**Fig 14:**
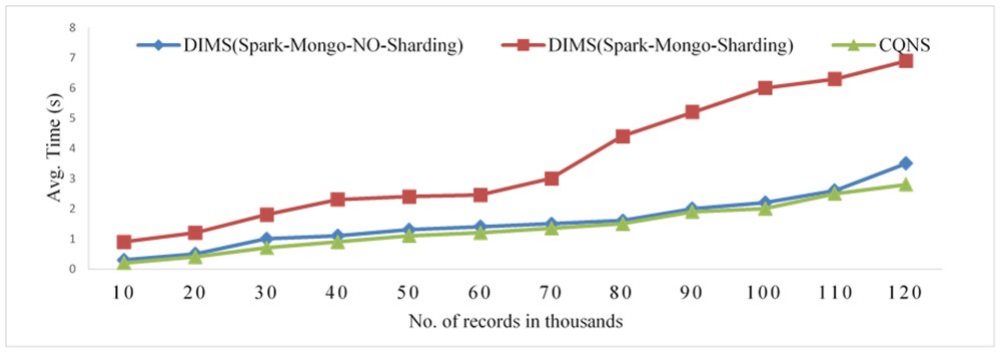
Comparison of the average time using mongo import and apache spark with Sharding and no-Sharding and CQNS.

**Fig 15:**
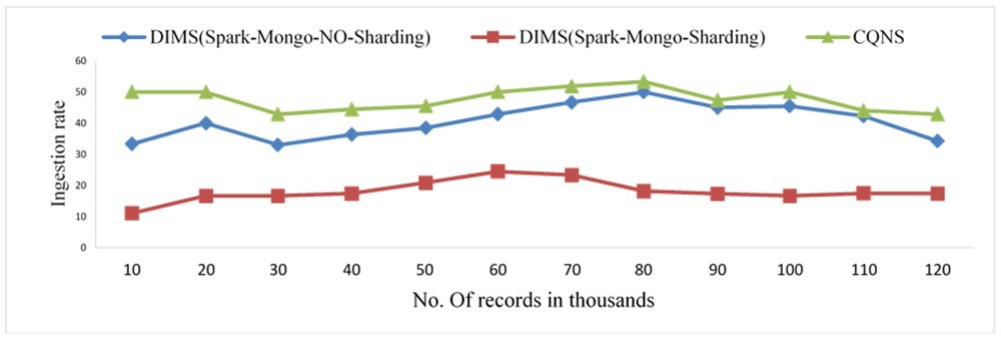
Comparison of the ingestion rate using dims mongo import, dims apache spark and CQNS.

An adaptive schema is proposed to improve the performance and time of execution of queries, taking into consideration the size of the research area. Figs 14 and 15 show and are extremely important, experiments which maximize the work of an integrated data store, particularly when this approach is utilized to execute very large quantities of data in addition to parallelism. The significance of merging the previous stages to obtain cost optimization is also proved in these experiments. The greater the number of records increased, the more the MongoDB Sharding efficiency decreased, compared with that of the No-Sharding databases, as shown in Fig 14, and this decreased efficiency influenced the execution time. By comparing Spark core and Spark SQL, the experiments found that the former differs from the latter because Spark SQL is loaded first. There is an increase in time, as shown in Fig 15.

### Experiments on Cassandra DB

**Fig 16:**
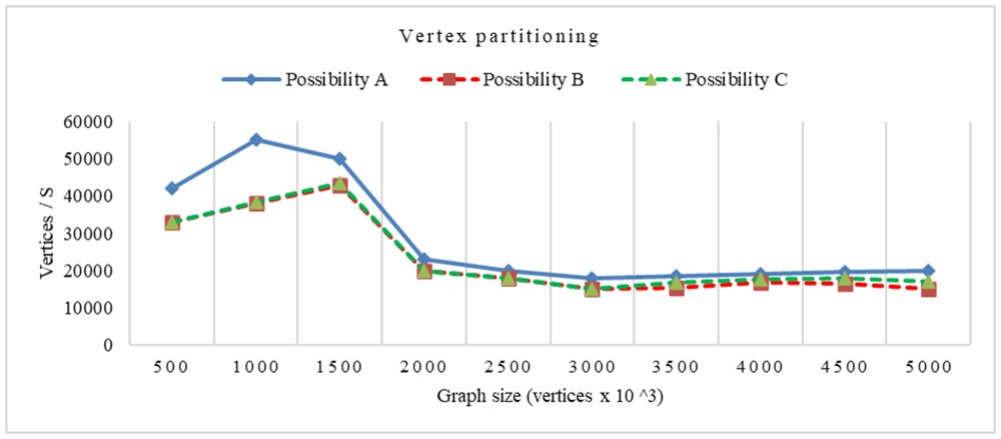
Latency time using vertex partitioning.

**Fig 17:**
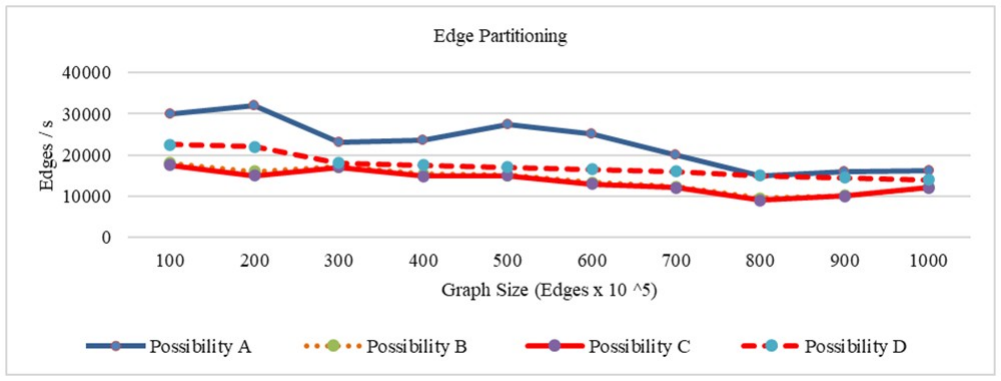
Latency time using edge partitioning.

The latency of read and write operations for vertex partitioning and edge partitioning when varying the number of nodes, is compared in the same from the last two experiments, it is obvious that among the compared systems, the CQNS framework, when dealing with the Cassandra dataset with partitioning, achieves the best latency and throughput values compared with those without using partitioning. environment of testing for possibilities 1, 2 and 3. The results for workloads A and C are shown in Figs 16 and 17, respectively.

### Performance variables of CQNS cassandra before partitioning and after partitioning on linear joins

**Fig 18:**
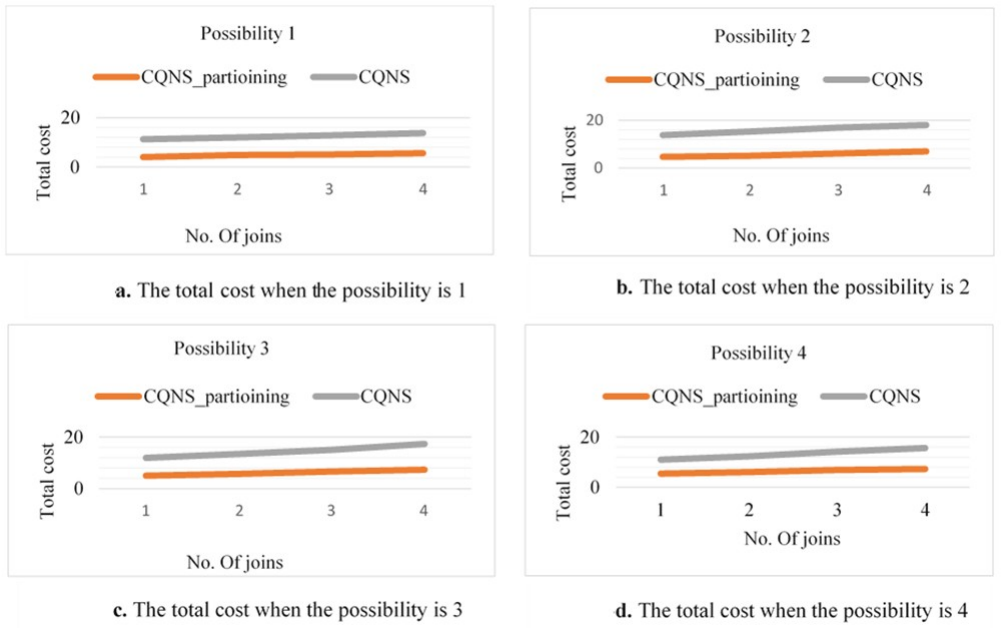
Comparison of the total cost using CQNS in Cassandra with edge partitioning and CQNS without partitioning on linear joins.

The results of the CQNS Framework on Linear Joins querying when dealing with the Cassandra dataset with segmentation obtain the best latency and throughput values, compared to without using segmentation in Linear Joins as shown in Fig 18a–18d.

### Performance variables for cassandra compared with those for MongoDB on linear joins

**Fig 19:**
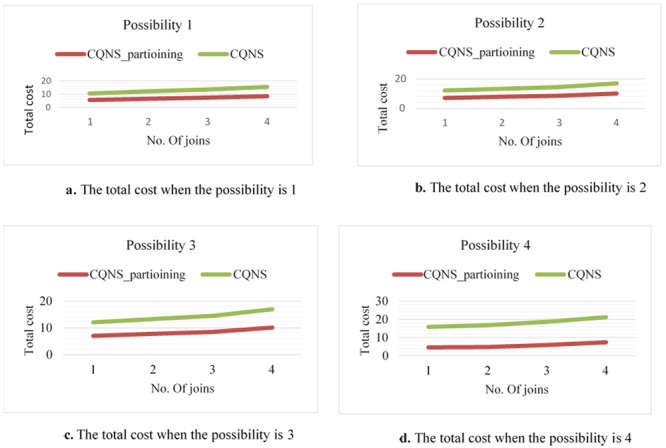
Comparison of the total cost using CQNS in CASSANDRA with edge partitioning and CQNS without partitioning on star joins.

The results of the CQNS Framework on star Join’s querying, when dealing with the Cassandra dataset with segmentation, obtain the best latency and throughput values, compared to without using segmentation in star Joins as shown in Fig 19a–19d.

### Performance variables for Cassandra compared with those for MongoDB on linear joins

**Fig 20:**
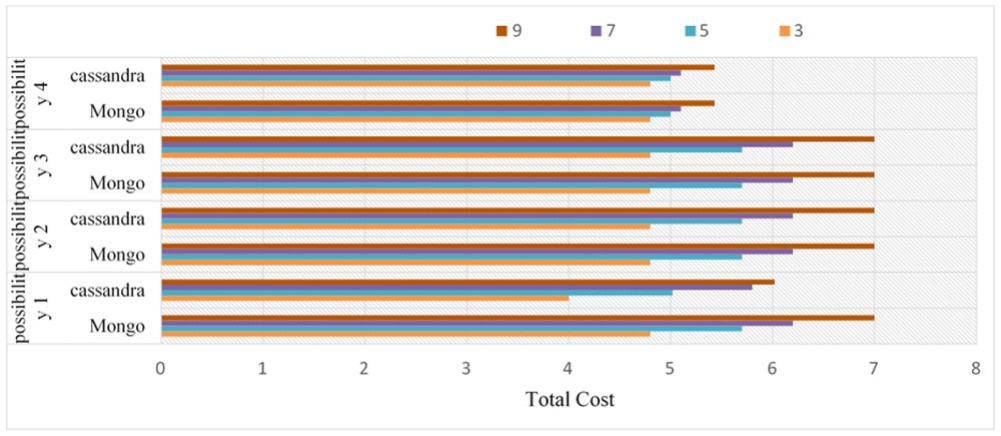
Performance variables for Cassandra compared with those for MongoDB on linear joins.

**Fig 21:**
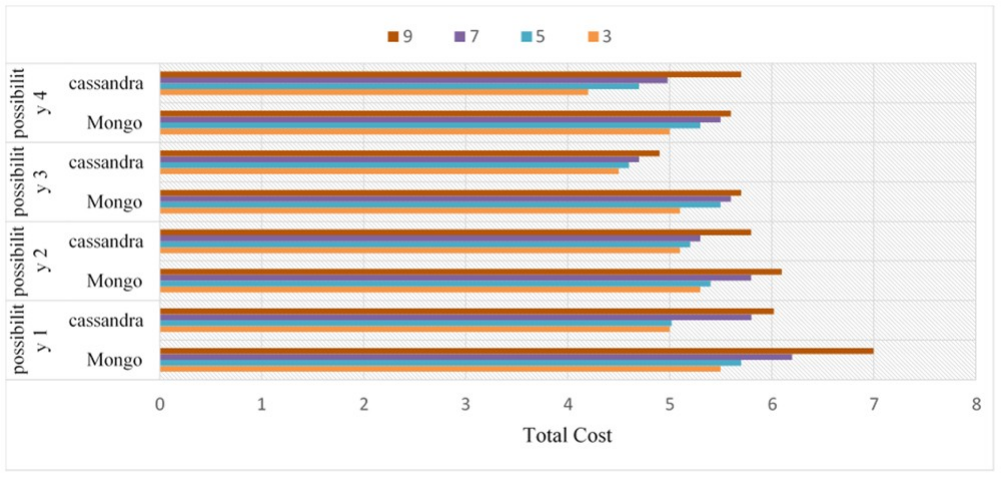
Performance variables for Cassandra compared with those for Mongo DB on star joins.

In this experiment, the performance of the techniques was evaluated by using partitioning on Cassandra using more than a certain number of joins from the "linear or star joins statements" feature using different numbers of joins. The experiment was performed for 75% more of the operations of Cassandra than those of MongoDB. Therefore, this study studied the different operations with respect to accuracy and memory. It is possible to reduce and manufacture this problem by separating these experiments 60 times into different nodes to give a more objective assessment of how to perform this evaluation. Consequently, thanks to a 10-fold cross-validation method, one dataset is shared in the two trial phases. Again, the research paper can see that the Cassandra and Mongo databases have increased performance effectiveness. These results are consistent with the results obtained in the first experiment. In short, Cassandra and MongoDB developers reach performance rates higher than 94.9% for all datasets in the two experiments, confirming the strength of the Cassandra database. The results for Cassandra and MongoDB on the two kinds of joins, linear and star joins, for the four possibilities are shown in Figs 20 and 21, respectively.

### Performance and cost over CQNS and Haery framework

**Fig 22:**
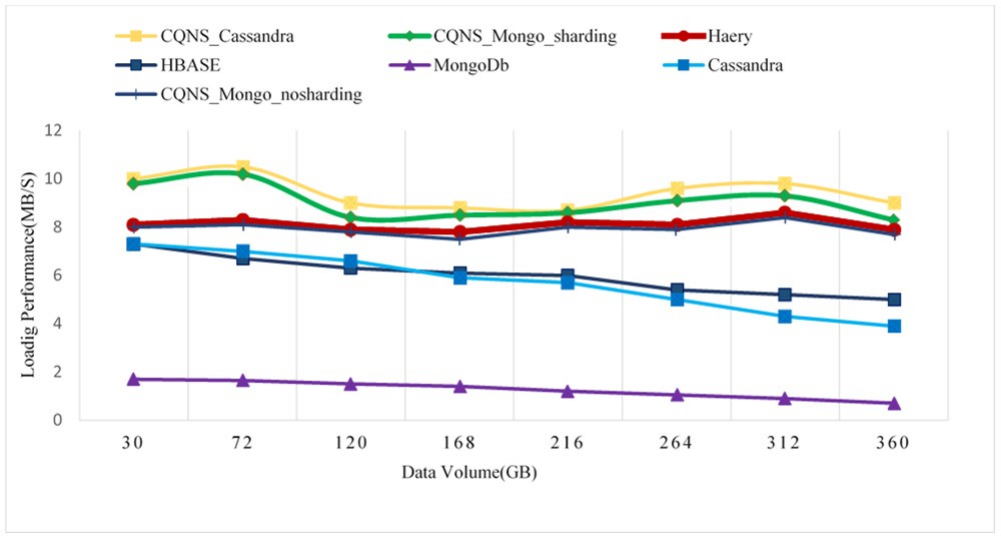
Comparison of loading speed among six data stores.

Since CQNS writes data in HDFS directly without writing data, Fig 22 shows the average download speed for HBase, Cassandra, MongoDB, CQNS with Cassandra and CQNS with Mongo on data sets of different data sizes. In the download experience, CQNS’s upload performance averaged 0.2, 10.2, 1.7, and 20.23 times higher than HBase, Cassandra, MongoDB, and Haery, to continue. the Cassandra with CQNS framework achieved better results than Haery [11], but the results of Haery using Mongo database are relatively better than CQNS results with no Sharding. On the other hand, when CQNS applied the Sharding technique, the results obtained are better than results obtained from Mongo and Cassandra databases, when using a large size of data.

## Results analysis and discussion

In this section, the core algorithms of CQNS and the results are evaluated, and compared with some popular NoSQL and relational databases using generated datasets and various query workload. The experiments conducted provided a comparison between CQNS and ODBPI to measure the cost time based on the number of joins. The results obtained, when using the Hadoop and spark, reflects higher performance compared to recent algorithms. CQNS evaluation implemented two types of joins: linear join and star join. And the results obtained from linear joins proved that they are better than star joins results. In addition, a comparison with the DIMS framework to measure the average time and ingestion time have been implemented using two different databases: Mongo and Cassandra databases. The CQNS results when using Mongo with Sharding technique is better than using Mongo without Sharding technique, especially when the amount of the data is huge. When comparison was done on the Cassandra database, it got better results when using the portioning technique than using it without portioning. This study also compared two types of partitioning in Cassandra database. According to CQNS experiments the edge portioning has got better results than vertex, especially when using a large size of data. When adding the comparison process with Haery framework, the Cassandra with CQNS framework achieved better results than Haery, but the results of Haery using Mongo database are relatively better than CQNS results with no Sharding. On the other hand, when CQNS applied the Sharding technique, the results obtained are better than results obtained from Mongo and Cassandra databases, when using a large amount of data.

## Conclusions and future work

It is difficult to perform complex queries across many data stores. Unlike applications that use a single data store, there is a request for additional efforts. This paper introduced a framework to handle complex query database. This framework consists of three stages. The first of which is responsible for defining the database engine that id matched with the user query sentences. In second stage the system sends user queries to a processing stage containing Hadoop HDFS to store data, the k-aggregation algorithm with MapReduce. The last stage either works with the SQL engine or the selected NoSQL Engine to do the job required. It should be noted that firstly, a vector holding the names of the SQL and NoSQL Engine is created to help in defining the database engine matched with the user query. This paper proposes a time model for calculating time cost and therefore it used Sharding technology with Mongo database queries to segment data and reduce the time used to query. On the other hand, this study used two types of partitioning in Cassandra database, one of them is the Edge and the second is Vertex. There is an intention to develop a future plan that will improve performance of the revised approach and add libraries for more databases to suit the needs of the different users. Moreover, other types of NoSQL Databases such as Sybase, Oracle, Access and other NoSQL Databases may be considered to demonstrate and expand the proposed framework.
